# Hijack it, change it: how do plant viruses utilize the host secretory pathway for efficient viral replication and spread?

**DOI:** 10.3389/fpls.2012.00308

**Published:** 2013-01-11

**Authors:** Camilo Patarroyo, Jean-François Laliberté, Huanquan Zheng

**Affiliations:** ^1^Department of Biology, McGill UniversityMontreal, QC, Canada; ^2^INRS-Institut Armand-Frappier, Institut National de la Recherche ScientifiqueLaval, QC, Canada

**Keywords:** ER, Golgi, endosomes, virus replication, virus transport

## Abstract

The secretory pathway of eukaryotic cells has an elaborated set of endomembrane compartments involved in the synthesis, modification, and sorting of proteins and lipids. The secretory pathway in plant cells shares many features with that in other eukaryotic cells but also has distinct characteristics important for fundamental cell and developmental processes and for proper immune responses. Recently, there has been evidence that the remodeling of this pathway, and often the formation of viral-induced organelles, play an important role in viral replication and spread. The modification of the host secretory pathway seems to be a common feature among most single-stranded positive ss(+)RNA and even some DNA viruses. In this review, we will present the recent advances in the understanding of the organization and dynamics of the plant secretory pathway and the molecular regulation of membrane trafficking in the pathway. We will also discuss how different plant viruses may interact with the host secretory pathway for their efficient replication and spread, with a focus on tobacco mosaic virus and turnip mosaic virus.

## INTRODUCTION

Like any other eukaryotic cells, plant cells are characterized by an elaborated secretory pathway composed of a complex network of organelles including the endoplasmic reticulum (ER), the Golgi apparatus, the *trans*-Golgi network (TGN), various endosomes, and vacuoles (Hanton et al., 2005; Bassham et al., 2008). This pathway is involved in the synthesis, modification and transport of proteins, lipids and polysaccharides (Bassham et al., 2008). Proteins made in the ER can be transported, via Golgi, toward the plasma membrane; on the other hand, proteins outside of the cells can also be internalized, via endocytosis. The proper organization and the dynamics of the secretory pathway are vital for normal cell development and physiology (Hanton et al., 2005).

In animal cells, it is known that efficient replication of many viruses involves a modification of the secretory pathway in host cells that generates a membrane structure designated as virosome, virus inclusion, virus factory, or viroplasm (for review, see Novoa et al., 2005; Netherton et al., 2007; Miller and Krijnse-Locker, 2008). Most animal viruses are membrane enveloped and it is known that the modification of the secretory pathway in host cells is also crucial for viral assembly (Netherton et al., 2007), intracellular and intercellular movement of viral complexes (Brandenburg and Zhuang, 2007), and inhibition of cellular secretion to reduce host immune response (Netherton et al., 2007). Different animal viruses modify the secretory pathway in many different ways for efficient viral replication, assembly, and spread along the secretory pathway (Netherton et al., 2007). For example, picornaviruses modify the early secretory pathway for RNA replication and inhibition of cellular secretion by interfering with the function of Arf1 and its guanine nucleotide exchange factor GBF1 in the formation of COPI vesicles (Hsu et al., 2010), while Norwalk virus inhibits cellular secretion through an interaction with COPII vesicles (Sharp et al., 2010). It is interesting that, although much less is known about how plant viruses modify the plant secretory pathway, recent data indicate that many plant viruses also utilize the plant secretory pathway for efficient replication and probably for cell-to-cell spread (Laliberte and Sanfacon, 2010; Schoelz et al., 2011; Verchot, 2011).

In this review, we will first highlight the recent progress in the organization and dynamics of the plant secretory pathway, we will then present the recent findings on how plant viruses may utilize the host secretory pathway for replication and intracellular and intercellular transport, with a focus on the recent advances on how the secretory pathway may be utilized by tobacco mosaic virus (TMV) and turnip mosaic virus (TuMV) for successful virus infection.

## THE ORGANIZATION AND DYNAMICS OF THE ER–GOLGI INTERFACE IN PLANT CELLS

The ER is an extensive membrane network that extends throughout the cytoplasm. The ER is the largest membranous structure in the eukaryotic cell; it is the site where proteins and lipids are synthesized and modified. The Golgi apparatus, with *cis*-Golgi facing the ER and *trans*-Golgi away from the ER, is the central station in the secretory pathway. In Golgi, proteins and lipids received from the ER will be further modified and sorted to the proper destination in the secretory pathway. Despite the fact that in animal cells the ER is mainly aligned with microtubules (Du et al., 2004) and in plant cells with microfilaments (Boevink et al., 1998), the morphology of the ER in plants is quite similar to that in animal cells. The ER in both mature animal and plant cells exhibits a labyrinth-like morphology composed of membranous tubules and cisternae (Hu et al., 2009; Orso et al., 2009; Chen et al., 2011, 2012). In addition, the mechanisms that mediate the tubulation of the ER (Voeltz et al., 2006; Sparkes et al., 2010) as well as the generation of interconnected ER network also appear to be conserved across different eukaryotic cells (Hu et al., 2009; Orso et al., 2009; Chen et al., 2011; Zheng and Chen, 2011). On the other hand, the organization of the Golgi apparatus is quite different between mammalian and plant cells. In mammalian cells, Golgi stacks form a single large perinuclear ribbon at the microtubule organization center peripheral to the nucleus (Ladinsky et al., 1999); in plant cells, the Golgi apparatus is present in the form of numerous individual cisternal stacks that move rapidly along the actin/ER cable in the cytoplasm (Boevink et al., 1998). As such, the morphology of the protein transport in the ER–Golgi interface appears very different between mammalian and plant cells.

Transport of proteins from the ER to the Golgi apparatus in both animal and plant cells starts from a transitional ER domain called ER export site (ERES). In mammalian cells, the ERESs are relatively stationary (Hammond and Glick, 2000), which are linked with the *cis*-Golgi by ERGIC, an ER–Golgi intermediate compartment made by the fusion of COPII vesicles formed at the ERESs (will be discussed below). In plant cells, individual ERESs are believed to be motile and tightly associated with Golgi stacks (daSilva et al., 2004). The ERES works together with individual Golgi stacks as a single functional unit (daSilva et al., 2004); no intermediate ERGIC has been revealed in plant cells. Yet it appears that the molecular mechanisms underlying protein transport in the ER–Golgi interface are very conserved between animal and plant kingdoms (Lee and Miller, 2007; Hsu and Yang, 2009; Hwang and Robinson, 2009; Marti et al., 2010; Chen et al., 2012).

In animal cells, transport from the ER to the Golgi apparatus is mediated by COPII vesicles (Lee and Miller, 2007). The proteins required for the formation of COPII vesicles from the ER include small Sar1 GTPase, membrane anchored **g**uanine **e**xchange **f**actor (GEF) Sec12, Sec23/Sec24 heterodimers for cargo selection and initiation of ER membrane curving (Bickford et al., 2004), and Sec13/Sec31 for the final formation of cage-like spherical vesicle (Lee and Miller, 2007). The fusion of COPII vesicles to generate ERGIC, which will further mature into *cis*-Golgi cisternae, requires small Rab1 GTPase and ER-localized SNARE (**s**oluble ***N***-ethylmaleimide-sensitive-factor **a**daptor-protein **re**ceptors) proteins (Bonifacino and Glick, 2004). In plant cells, despite the morphological difference in the ER–Golgi interface, all these key proteins exist (Bassham et al., 2008; Marti et al., 2010). It has been experimentally demonstrated that AtSEC24a as well as Rab-D proteins, homologs of Rab1, are required for ER-to-Golgi transport in plant cells (Batoko et al., 2000; Faso et al., 2009; Qi and Zheng, 2011). In mammalian cells, the retrograde Golgi transport, essential for recycling of proteins and lipids back to the ER in order to maintain equilibrium with the COPII-dependent transport, occurs mainly through COPI vesicles (Hsu and Yang, 2009). The COPI coatomer is composed by two protein complexes, F- and B-COP. The formation of COPI vesicles in Golgi cisternae requires small Arf1 GTPase, whose activity is regulated by Sec7-domain Arf1-GEFs (Hsu and Yang, 2009). Brefeldin A (BFA), a fungal metabolite, has been found to be a specific inhibitor that interferes with the interaction between Sec7-domain Arf-GEF and Arf proteins (Nebenfuhr et al., 2002). In *Arabidopsis thaliana*, electron tomography analysis have shown the existence of two types of COPI coated vesicles, the COPIa vesicles derived from *cis*-Golgi and the COPIb vesicles derived from the *trans*-Golgi (Hwang and Robinson, 2009).

## THE ORGANIZATION AND DYNAMICS OF POST-GOLGI TRAFFIC NETWORK IN PLANT CELLS

Proteins and lipids, after being transported and modified in the Golgi apparatus, are further transported to either the plasma membrane, or to the lysosome/vacuole (in plant cells). In mammalian cells, cargo molecules modified in the Golgi apparatus are usually delivered to the TGN where they are sorted into either secretory vesicles that move to the plasma membrane, or clathrin-coated vesicles to deliver cargoes to the endosomes and then the lysosome (Traub and Kornfeld, 1997). This anterograde protein transport to the plasma membrane is usually balanced by various clathrin-coated vesicle mediated endocytic pathways (Gruenberg and Maxfield, 1995; Clague and Urbe, 2001). After being formed in the plasma membrane, clathrin-coated endocytic vesicles are first delivered to the early endosome (EE), a compartment that can be marked by Rab5, a small GTPase required for the fusion of clathrin-coated vesicles to the EE (Gorvel et al., 1991; Rink et al., 2005). At the EE, proteins can be either recycled back to the plasma membrane via recycling endosomes (RE; Grant and Donaldson, 2009), or further sorted to the late endosomes (LE) and the lysosome. It is thought that the protein recycling at the EE via the RE is mediated by Rab11 (Grant and Donaldson, 2009). The LE is believed to mature by the fusion of different EEs mediated by small Rab7 GTPase and LE-specific SNARE proteins (Bonifacino and Glick, 2004; Rink et al., 2005). At the LE, ESCRT (the endosomal sorting complexes required for transport) at the surface can selectively pick ubiquitinated membrane proteins to be directed to the lysosomes for further degradation (Katzmann et al., 2001).

In plant cells, initially the TGN was thought to be absent, but recent evidence suggests that the TGN may be a motile organelle derived from the Golgi apparatus (Dettmer et al., 2006; Kang et al., 2011; Qi et al., 2011). It has been demonstrated that the plant TGN could act as a sorting station and simultaneously release two types of vesicles: secretory vesicles heading to the plasma membrane and the cell wall (Preuss et al., 2006; Szumlanski and Nielsen, 2009; Qi et al., 2011), and clathrin-coated vesicles mediating transport to vacuoles (Kang et al., 2011). The organization of the dynamics of the post-Golgi traffic network in plant cells is, however, not well studied. It appears that protein transport in the plant post-Golgi traffic network is quite distinct from that in mammalian cells. There are several lines of evidence suggesting that the TGN in plants also serves as an EE (Dettmer et al., 2006; Qi et al., 2011). At the TGN/EE, proteins can be either recycled back to the plasma membrane (Ueda et al., 2001), or further transported to multi-vesicular bodies (MVB) or prevacuolar compartments (PVC), a compartment equivalent to the LE in animal cells (Spitzer et al., 2009). In plant cells, no RE has been identified. Different from mammalian cells, in plant cells the TGN/EE is highlighted by the presence of different Rab-A proteins (Szumlanski and Nielsen, 2009; Kang et al., 2011; Qi et al., 2011), homologs of the animal Rab11 protein; the MVB/PVC is marked by Rab-F proteins (Ueda et al., 2001), homologs of the Rab5 in animal cells. MVB/PVC bearing ESCRT that can selectively pick ubiquitinated membrane proteins has also been reported (Spitzer et al., 2009).

## THE ROLE OF THE HOST SECRETORY PATHWAY IN PLANT VIRAL REPLICATION

Viruses are obligate intracellular parasites that depend on cellular materials for their replication and spread. As mentioned in the introduction, replication and assembly of many membrane enveloped animal viruses occur in an intracellular compartment termed virus factory, viral inclusion, viroplasm, or virosome whose generation requires the modification of the host secretory pathway (Novoa et al., 2005; Netherton et al., 2007; Miller and Krijnse-Locker, 2008). Despite the fact that most plant viruses are not membrane enveloped, recent research revealed that plant viruses also utilize the host secretory pathway for viral replication (Laliberte and Sanfacon, 2010; Verchot, 2011).

Studies with TMV, an extensively studied member of *tobamoviruses*, showed that TMV infection causes severe modifications of the ER, converting it into large irregular aggregates early in infection (Reichel and Beachy, 1998). This ER-derived structure contains virus particles, the virus-encoded 183 and 126 kDa replicases, movement protein (MP), vRNA, ribosomes, and host translation elongation factor EF1-α (Beachy and Zaitlin, 1975; Heinlein et al., 1998; Reichel and Beachy, 1998; Mas and Beachy, 1999; Figueira et al., 2002). Therefore it is believed that this ER-derived structure is the site for TMV replication, which is often termed as viral replication complex (VRC; Heinlein et al., 1998). However, how exactly these TMV replication complexes are formed is not yet clear. The MP and the replicase components 126 and 183-kDa proteins are associated with the ER (Reichel and Beachy, 1998; Hagiwara et al., 2003). The MP, when expressed ectopically, can induce modifications in the ER (Reichel and Beachy, 1998) and the 126-kDa replicase, when expressed ectopically, can also induce the formation of cytoplasmic inclusion bodies (Ding et al., 2004). Therefore it is possible that these viral proteins are involved in the formation of TMV replication complexes. Furthermore, the TMV replication complexes are found to be associated with the host transmembrane proteins TOM1 (Yamanaka et al., 2000; Hagiwara et al., 2003) and ARL8, a small GTP-binding protein (Nishikiori et al., 2011). Both proteins are required for efficient TMV RNA replication (Nishikiori et al., 2011). This suggests that host proteins also play important roles in the formation of TMV replication complexes in the secretory pathway. It will be interesting to study how exactly these viral and host proteins are involved in the process.

In the case of *potyviruses*, TuMV infection induces the formation of small vesicular compartments that move rapidly along the microfilaments and the ER (Beauchemin et al., 2007; Cotton et al., 2009; Grangeon et al., 2012). The polyprotein 6K_2_-VPg-Pro, through its hydrophobic 6K_2_ domain, is responsible for the formation of these motile structures (Beauchemin et al., 2007). The presence of viral dsRNA, an obligate intermediate in the replication of the ssRNA viruses, as well as the host eukaryotic initiation factor iso4E (eIF(iso)4E), poly-A binding protein, heat shock cognate 70-3 protein (Hsc70-3), and the eukaryotic elongation factor 1A (eEF1A; Beauchemin and Laliberte, 2007; Beauchemin et al., 2007; Dufresne et al., 2008; Thivierge et al., 2008; Cotton et al., 2009) in the 6K_2_-induced vesicles suggests that the viral genome replication takes place in these structures (Cotton et al., 2009). Interestingly, in cells infected by the *potyvirus* tobacco etch virus (TEV), the motile 6K_2_ vesicular compartments are ER-derived and are associated with ERESs. The formation of the structure is COPII- and COPI-dependent (Wei and Wang, 2008). The motile 6K_2_ vesicles in TuMV-infected cells are associated but not perfectly with COPII vesicles as well as Golgi stacks (Grangeon et al., 2012). BFA can enhance the association of 6K_2_ vesicles with COPII and Golgi stacks (Grangeon et al., 2012). The interpretation of these facts is that the formation as well as the transport (will be discussed further) of the motile 6K_2_ structures requires an interaction between 6K_2_ and host proteins involved in the formation and transport of COPII and COPI vesicles.

Recently, we reported that TuMV induces the formation of a perinuclear globular structure with an amalgamation of viral 6K_2_, ER, Golgi, and COPII membranes as well as the presence of chloroplasts within the structure (Grangeon et al., 2012). This globular structure maintains a dynamic connection with the cortical ER and the Golgi apparatus; additionally the motile 6K_2_ vesicles may originate from the globular structure (Grangeon et al., 2012). We hypothesize that this globular structure could provide an extended platform for viral replication and protein synthesis (Grangeon et al., 2012). Indeed, the formation of large perinuclear ER-derived structures has also been observed in several other plant viruses. For example, cowpea mosaic virus (CPMV) induces the proliferation of ER membranes (de Zoeten et al., 1974; Carette et al., 2000, 2002a) that are often next to the nuclei. By the incorporation of H^3^-uridine in the infected leaves, newly synthesized viral RNA can be found in the perinuclear membranous structures induced by CPMV (de Zoeten et al., 1974). Grapevine fanleaf virus (GFLV) induces the formation of a punctate-spongy perinuclear structure composed of reorganized ER membranes and active synthesis of RNA is found in these ER-derived structures (Ritzenthaler et al., 2002). Potato virus X (PVX) also forms an ER-contained perinuclear structure called X-body (Tilsner et al., 2012) where the viral replicase and TGB1 are located.

The exact mechanism by which these globular structures are formed remains unclear. It is known in plant cells that disruption of the formation of COPII vesicles also induces the formation of perinuclear clusters of the ER and Golgi membranes (Faso et al., 2009). The 60K helicase of CPMV interacts with the ER localized v-SNARE-like protein VAP27 in the proliferation of ER membranes (Carette et al., 2002b), suggesting that an interaction of viral and host ER proteins may be required in the process. It is interesting, however, in TuMV infected cells, the BFA treatment does not inhibit the formation of the perinuclear globular structure nor viral replication (Grangeon et al., 2012). Although the perinuclear structure in TuMV-infected cells contains the ER and Golgi membranes, it seems that the formation of the structure does not require a functional early secretory pathway. Similarly, BFA seems not affect replication of melon necrotic spot virus but it may have a negative impact on viral cell-to-cell movement (Genoves et al., 2010). On the other hand, in GFLV-infected cells, although it is not known if the BFA treatment inhibits the formation of the perinuclear structure, such treatment results in a significant reduction of the replication efficiency of the virus (Ritzenthaler et al., 2002). It is highly likely that different viruses use different strategies to generate membranous structures for their efficient replication.

In animal cells, the generation of the virus factory containing the membranous structures of the host secretory pathway is also thought to be a mechanism for viruses to hide from host cell anti-viral defenses (Novoa et al., 2005; Miller and Krijnse-Locker, 2008). With regards to this, it is interesting to note that in *N. benthamiana* cells expressing a dominant-negative Vps23, Vps24, Snf7p, and Vps4p, components of ESCRT complexes, the replication of the tomato bushy stunt virus (TBSV), a member of *tombusvirus*, is impaired (Barajas et al., 2009). In addition, in yeast *vps23δ* or *vps24δ* cells, the activity of the viral replicase p92 is reduced and the (-) stranded viral RNA is more accessible to ribonucleases (Barajas et al., 2009). How the absence of ESCRT complexes in host cells could lead to a reduced activity of p92 and less stable viral RNA remains unknown. As mentioned previously, the ESCRT complexes are required for the recognition and sorting of ubiquitin-modified cargo proteins into MVBs and eventually to the vacuole (Katzmann et al., 2001; Spitzer et al., 2009). It is possible that ESCRT complexes are recruited into the viral replicase complex at some stages to help the virus to evade the recognition by host defense proteins and/or to avoid the degradation by the gene silencing machinery. Indeed, the p33 replication protein of TBSV interacts with Vps23p (Barajas et al., 2009), suggesting that the viral protein may be involved in the recruitment of ESCRT into the virus factory, or regulate the activity of ESCRT in the factory.

Although most plant viruses are not membrane enveloped, it is interesting to note that there is a small group of plant viruses such as the tomato spotted wilt virus (TSWV) that are membrane enveloped (Mumford et al., 1996). TSWV is a plant-infecting counterpart of the animal infecting viruses within the arthropod-borne Bunyaviridae. It is known that *bunyaviruses* that infect animal cells are replicated in tubular viral factories that are built around Golgi stacks but are connected to both mitochondria and the rough ER (Walter and Barr, 2011). After the final virion assembly in the lumen of swollen Golgi stacks, viruses bud into secretory vesicles in which they migrate toward the plasma membrane and are then secreted (Walter and Barr, 2011). However, it is not clear if this is also the case for TSWV. TSWV is replicated in infected plant cells in association with a membrane modification of the ER and Golgi (Ribeiro et al., 2008). The viral particles are assembled within the infected plant cells when ribonucleoprotein complexes (RNPs) are enwrapped by Golgi cisternae. The Golgi membrane wrapped RNPs then fuse with each other and with ER membranes, producing a singly enveloped vesicle where virus particles are retained until uptake by its insect vector (Kitajima et al., 1992; Kikkert et al., 1999). It was recently shown that the TSWV glycoproteins Gn and Gc induce the formation of ER- and Golgi-derived membranous structures upon ectopic expression in plant protoplasts (Ribeiro et al., 2008). The Gn glycoprotein localizes to both the ER and Golgi and is able to redirect the ER-arrested glycoprotein Gc to Golgi in a COPII-dependent manner upon co-expression (Ribeiro et al., 2009). In addition, both Gn and Gc interact with the TSWV nucleocapsid protein (N) and RNPs in ER and Golgi (Ribeiro et al., 2009). Therefore it seems that viral Gn and Gc glycoproteins, transported within the ER and Golgi, play an important role in the generation of the hybrid ER–Golgi structure where the virus is replicated and assembled. As one of the few plant viruses that are enveloped by lipid membranes, it will be interesting to examine how TSWV migrates, once assembled in the cytoplasmatic vesicles, to the neighboring cells. Likely, TSWV moves cell-to-cell differently from its animal counterpart, because the viral NSm protein, which was shown to restore the cell-to-cell and long distance movement of a movement-defective TMV clone (Lewandowski and Adkins, 2005), forms tubules in association with plasmodesmata (PD) upon TSWV infection of *N. rustica* (Storms et al., 1995).

## ROLE OF THE HOST SECRETORY PATHWAY IN INTRA- AND INTER-CELLULAR TRANSPORT OF VIRUSES

As described above, many plant viruses replicate in infected cells in association with membranous structures derived from the host secretory pathway (Laliberte and Sanfacon, 2010; Verchot, 2011). Consequently, virions or viral nucleic acid–protein complexes must travel from the site where they are replicated to the neighboring cells to start the replication cycle again in order to cause a systemic infection. It has been generally accepted that plant viruses move from an infected cell to its neighboring cells through PD (Schoelz et al., 2011; Ueki and Citovsky, 2011). However, the virions and even the naked genome of plant viruses are too large to fit through unaltered PD. To facilitate the spread of viral particles or replication complexes, most plant viruses encode at least one dedicated protein termed movement protein (Lucas, 2006) to modify the structure of PD (Lucas, 2006; Schoelz et al., 2011; Ueki and Citovsky, 2011).

How could viral particles or replication complexes generated in the virus factory move to PD? Studies of various plant viruses over the past decades indicated that host cytoskeletal elements provide viruses the means to move from their sites of replication to PD from where they travel to the neighboring cells (Schoelz et al., 2011; Ueki and Citovsky, 2011). However, recent studies of some plant viruses indicate that the host secretory pathway and protein transport machineries may also be hijacked by viruses for their efficient transport to PD and cell-to-cell movement. In TMV, the MP and the 126 kDa replicase proteins are required for its efficient movement (Reichel and Beachy, 1998; Hirashima and Watanabe, 2001). Both proteins as well as TMV replication complexes are found to be associated with the ER (Heinlein et al., 1998; Yamanaka et al., 2000; Hagiwara et al., 2003). However, it is interesting that lower concentrations of BFA (10 μg/ml) or overexpression of dominant negative Sar1 does not inhibit intercellular transport of neither the MP of TMV nor the TMV replication complexes to PD (Tagami and Watanabe, 2007), while a higher concentration of BFA (>50 μg/ml) did inhibit the movement of the TMV MP to PD (Heinlein et al., 1998; Wright et al., 2007). The higher concentration of BFA is known to disrupt interconnected ER network (Zheng et al., 2004). Thus the interpretation of the different effect of different BFA concentrations could be that the transport of TMV to PD requires a networked ER but does not require the COPII-/COPI-dependent molecular machinery. Considering the fact that ER tubules traverse through PD to the neighboring cells, it is possible that ER tubules are used directly by TMV as a transport conduit for both intra- and inter-cellular transport.

In the case of the members of the genus *Potyviruses*, it appears that intracellular transport of virus replication complexes from the site where they replicate to PD requires the classic COPII/COPI vesicle trafficking machinery in the secretory pathway. As described in the previous section, in both TuMV- and TEV-infected cells, motile 6K_2_ vesicular structures are identified as a site where viral genome can be replicated (Beauchemin et al., 2007; Cotton et al., 2009; Grangeon et al., 2012). Yet the formation of these motile 6K_2_ vesicles is COPII- and COPI-dependent (Wei and Wang, 2008). They are generated from ERESs, move to Golgi stacks, and are transported along the actin and the ER to the cell periphery (Beauchemin et al., 2007; Wei and Wang, 2008; Cotton et al., 2009; Grangeon et al., 2012). Furthermore, the movement of 6K_2_ and the cell-to-cell movement of TuMV can be inhibited by BFA at 10 μM (Grangeon et al., 2012). For *potyviruses*, the proteins VPg, CI, CP, and P3N-PIPO have been implicated in inter-cellular movement of viruses through PD (Nicolas et al., 1997; Rojas et al., 1997; Carrington et al., 1998; Wei and Wang, 2008; Wei et al., 2010). It is interesting that recently, Wei et al. (2010) demonstrated that the trafficking of P3N-PIPO and CI to PD requires a COPII-/COPI-dependent protein transport machinery, but not the actomyosin system (Wei et al., 2010) as the localization of P3N-PIPO and CI to PD is abolished in infected cells treated with BFA, or in cells expressing a mutant version of Sar1 [Sar1(H74L)], but not by actin disrupting agents. It seems that the transport of the *potyvirus* replication complex to the cell periphery requires the classic COPII/COPI vesicle trafficking machinery in the secretory pathway and the actomyosin system, but the transport of the viral proteins to PD for the subsequent modification of PD only requires the classic COPII/COPI vesicle trafficking machinery in the secretory pathway. However, NSvc4, the putative MP of rice strip virus (RSV) and MP17, the MP of potato leaf roll virus (PLRV), when expressed alone, are targeted to PD in COPII- and actin-dependent manners (Vogel et al., 2007; Yuan et al., 2011). Similarly, the MP protein p7B of *melon necrotic spot virus* is found to localize to the ER, Golgi, and PD when ectopically expressed (Genoves et al., 2010). Genoves et al. (2010) show that both BFA and latrunculin B inhibit the transport of p7B to Golgi and PD at the level the ER. However, it is generally accepted that in plant cells the actomyosin system is not required for protein transport from the ER to Golgi (Brandizzi et al., 2002).

As mentioned previously, in plant virus infected cells, PD has to be modified, either with an increase in SEL or formation of tubules (reviewed by Schoelz et al., 2011), to allow viral particles or replication complexes to spread. Recent data suggested that, in addition to movement-related proteins encoded by various viruses, some host proteins, for example, PDLP1, a type-I membrane protein located to PD, appear to be required for successful modification of PD (Huang et al., 2001; Thomas et al., 2008; Amari et al., 2010). When *Arabidopsis* plants expressing mutant versions of *PDLP1*, *PDLP2*, and *PDLP3* are inoculated with cauliflower mosaic virus (CaMV), a *Caulimovirus* that induces tubules through which CaMV virions move, significantly fewer plants present systemic infections (Amari et al., 2010). The replication of CaMV in the protoplasts carrying the triple mutation is, however, comparable to the replication in wild type *Arabidopsis* (Amari et al., 2010). This suggests that the PDLP1 protein plays a vital role in the movement of the virus, possibly in the modification of PD for the formation of tubules. It is interesting that the targeting of PDLP1 to PD is COPII-dependent (Thomas et al., 2008). However, in CaMV-infected cells, it appears that targeting of the MP of the virus to the cell periphery is not abolished by the treatment of BFA (Huang et al., 2000). It seems that transport of viral proteins of CaMV and host proteins to PD relies on different transport routes.

In addition to the above mentioned roles that the host secretory pathway may play in the transport of virus replication complexes and viral and host proteins required for efficient spread of viruses, it is worth noting that the TGB2 protein of potato mot top virus (PMTV), which is located to both the ER and PD and is probably involved in the modification of PD for intercellular virus movement (Cowan et al., 2002), can also be found in vesicles marked by FM4-64 (Haupt et al., 2005), a fluorescent dye that is widely used to track endocytosis in plant cells (Qi et al., 2011). TGB2 interacts with a J-domain chaperone essential for the endocytic protein transport and is also co-localized with Ara7, a Rab-F protein localized to the PVC/MVB (Haupt et al., 2005). In addition to this, the MP of TMV and a DNA virus, cabbage leaf curl virus (CaLCuV), physically interact with the clathrin-associated SNARE-interacting protein synaptotagmin (Lewis and Lazarowitz, 2010). Synaptotagmin is a Ca^2^^+^ sensor and down-regulation of this protein inhibits endocytosis and intercellular spread of TMV and CaLCuV (Lewis and Lazarowitz, 2010). However, the exact role that this endocytic protein transport pathway may play in the virus multiplication is yet to be determined. Endocytosis is known to be a mechanism for cells to regulate the localization and/or function of some plasma membrane localized proteins (Gruenberg and Maxfield, 1995), perhaps the accumulation and function of proteins required for modification of PD for efficient virus spread needs to be regulated by endocytosis.

## CONCLUDING REMARKS

Plant cells have a sophisticated secretory pathway that is vital for their growth and survival in response for prevailing environmental conditions. In recent years, there has been an explosion of information regarding the organization of the plant secretory pathway and the molecular mechanisms that regulate protein transport in the plant secretory pathway. For successful infection of host plant cells, it seems that plant viruses have developed different yet highly host-adapted strategies to take advantage of the molecular machineries provided by the host secretory pathway to replicate and move from infected to uninfected cells. However, how plant viruses interplay with their host secretory pathway is not well understood. It is highly likely that different plant viruses interact differently with the host secretory pathway for efficient virus multiplication. To unravel the exact mechanisms by which different viruses are replicated, and transported to and through PD to neighboring cells, we need to identify more of the host factors required for the multiplication of different viruses. We need to understand how these host factors interact with different viral factors and to figure out the functional significance of these interactions in viral infection. We believe that a further understanding of these issues is important for those who wish to develop plant cultivars that resist to virus infection. On the other hand, such research could also serve as a way to unlock the secrets of the host secretory pathway in plant cells.

## Conflict of Interest Statement

The authors declare that the research was conducted in the absence of any commercial or financial relationships that could be construed as a potential conflict of interest.
